# Inhibition of complement pathway activation with Pozelimab, a fully human antibody to complement component C5

**DOI:** 10.1371/journal.pone.0231892

**Published:** 2020-05-08

**Authors:** Adrianna Latuszek, Yashu Liu, Olav Olsen, Randi Foster, Marc Cao, Irena Lovric, Ming Yuan, Nina Liu, Henry Chen, Qian Zhang, Hui Xiao, Carola Springer, George Ehrlich, Vishal Kamat, Ashique Rafique, Ying Hu, Pamela Krueger, Tammy Huang, William Poueymirou, Robert Babb, Michael P. Rosconi, Marc W. Retter, Gang Chen, Lori Morton, Brian Zambrowicz, Jingtai Cao, Carmelo Romano, William C. Olson

**Affiliations:** Regeneron Pharmaceuticals, Inc., New York, NY, United States of America; Hospital for Sick Children, CANADA

## Abstract

Complement is a key component of the innate immune system. Inappropriate complement activation underlies the pathophysiology of a variety of diseases. Complement component 5 (C5) is a validated therapeutic target for complement-mediated diseases, but the development of new therapeutics has been limited by a paucity of preclinical models to evaluate the pharmacokinetic (PK) and pharmacodynamic (PD) properties of candidate therapies. The present report describes a novel humanized C5 mouse and its utility in evaluating a panel of fully human anti-C5 antibodies. Surprisingly, humanized C5 mice revealed marked differences in clearance rates amongst a panel of anti-C5 antibodies. One antibody, pozelimab (REGN3918), bound C5 and C5 variants with high affinity and potently blocked complement-mediated hemolysis *in vitro*. In studies conducted in both humanized C5 mice and cynomolgus monkeys, pozelimab demonstrated prolonged PK and durable suppression of hemolytic activity *ex vivo*. In humanized C5 mice, a switch in dosing from in-house eculizumab to pozelimab was associated with normalization of serum C5 concentrations, sustained suppression of hemolytic activity *ex vivo*, and no overt toxicity. Our findings demonstrate the value of humanized C5 mice in identifying new therapeutic candidates and treatment options for complement-mediated diseases.

## Introduction

The complement system is a vital, evolutionarily conserved arm of the innate immune system that enhances (complements) the ability of antibodies and phagocytic cells to clear pathogens from an organism. Activation of complement on pathogen surfaces initiates a cascade of proteolytic events that result in the formation of complement component split products and complexes that act as proinflammatory signals, mediate phagocytosis by opsonization, or induce cell lysis [1–3].

The complement cascade can be initiated through three different pathways (classical, lectin, or alternative), but all may result in eventual cleavage of C5 by C5 convertase. C5 convertase is a protein complex containing the C3 cleavage product, C3b, and other complement components that are specified by the initiating pathway [2,3]. Cleavage of C5 by the C5 convertase produces C5a and C5b, which perform distinct effector functions. C5a is an anaphylatoxin and potent proinflammatory mediator [4,5]. C5b sequentially recruits complement components C6, C7, C8, and up to 18 molecules of C9 to form the membrane attack complex (MAC), a transmembrane pore that disrupts the integrity of the target cell’s membrane [6,7].

Overactivation of the complement system has the potential to cause uncontrolled inflammation and tissue damage [8,9]. As a result, complement contributes to morbidity in many diseases. In addition, dysfunction of the complement system directly underlies the etiology of several diseases, including paroxysmal nocturnal hemoglobinuria (PNH), atypical hemolytic uremic syndrome (aHUS), glomerulopathies, and CD55 deficiency with hyperactivation of complement, angiopathic thrombosis, and protein-losing enteropathy (the CHAPLE syndrome) [1]. Hemolysis is a shared hallmark for several of these diseases, and *ex vivo* inhibition of complement-mediated lysis of red blood cells is a clinically validated biomarker for C5-targeted therapeutics [10,11].

In this study, we describe the utility of humanized C5 mice in isolating and developing pozelimab (REGN3918), a fully human antibody isolated from Regeneron’s VelocImmune^®^ human antibody mouse platform [12]. REGN3918 binds human and monkey C5 with high affinity and blocks hemolysis induced by both the classical (CP) and alternative (AP) complement pathways. Following a single 15 mg/kg subcutaneous (SC) dose, REGN3918 displayed prolonged pharmacokinetics and pharmacodynamics in both *C5*^*hu/hu*^ mice and cynomolgus monkeys and blocked complement mediated hemolytic activity *ex vivo* for at least 35 days. Further, switching the dosing of *C5*^*hu/hu*^ mice from in-house eculizumab to REGN3918 normalized serum C5 concentrations, and maintained suppression of hemolytic activity *ex vivo* in the absence of overt toxicity. Pozelimab may offer an alternative therapeutic option for patients suffering from PNH and aHUS, as our PK/PD results suggest that it may require less frequent dosing, normalize serum C5 levels and be efficacious in patients that carry rare C5 variants.

## Results

### Generation of high-affinity anti-C5 antibodies that block complement-mediated hemolysis *in vitro*

A human C5 immunization campaign using VelocImmune^®^ mice was undertaken to generate fully human anti-C5 antibodies that block terminal complement pathway activation. The kinetic properties of purified anti-C5 antibodies binding to human, monkey and mouse C5 were determined using SPR-Biacore technology at 37°C. A group of 6 antibodies were identified that bound specifically and with similarly high affinity to human C5 (KD = 262 pM to 6.27 nM) and monkey C5 (KD = 1.67 to 8.88 nM) but did not bind mouse C5 under the same experimental conditions (Table 1).

**Table 1:** Summary of kinetic properties for anti-C5 antibodies binding to human and cynomolgus monkey C5 at 37°C, pH7.4 and blocking CP- and AP-mediated hemolysis in normal human and cynomolgus monkey serum *ex vivo*.

Ant-hC5 Antibody	Binding to Human C5		Binding to Monkey C5		CP Human		CP Monkey		AP Human		AP Monkey	
	KD [M]	T_1/2_ (min)	KD [M]	T_1/2_ (min)	IC 50 [M]	% Block	IC 50 [M]	% Block	IC 50 [M]	% Block	IC 50 [M]	% Block
REGN3918	2.62E-10	50	8.55E-09	6	2.8E-09	95.0	2.0E-08	94.2	2.6E-08	70.9	8.9E-09	98.6
H4H12161P	9.90E-10	101	5.56E-09	16	2.6E-09	93.7	2.8E-08	100.0	2.6E-08	56.1	7.8E-09	98.4
H4H12171P	6.27E-09	15	1.85E-09	11	2.3E-09	94.9	8.7E-09	92.9	1.6E-08	42.0	7.2E-09	98.8
H4H11686N	4.69E-10	74	8.88E-09	6	3.1e-09	97.4	1.4E-08	94.5	1.3E-08	76.2	9.0E-09	98.7
H4H12164P	3.23E-10	90	6.30E-09	10	2.1E-09	95.9	2.0E-08	94.7	1.5E-08	69.7	9.1E-09	98.7
H4H12170P	1.40E-09	28	1.67E-09	13	2.4E-09	98.2	8.2E-09	99.0	1.8E-08	81.1	7.0E-09	99.2

Abbreviations: AP = alternative pathway; CP = classic pathway; KD = dissociation constant; T_1/2_ = half-life

To screen isolated antibodies for their ability to block the classical (CP) and alternative (AP) complement pathways, *ex vivo* hemolysis assays were used to assess the lysis of red blood cells in the presence or absence of anti-C5 antibody. CP-mediated hemolytic assays were performed by incubating hemolysin-sensitized sheep red blood cells with normal human or cynomolgus monkey serum at 37°C. Low nanomolar concentrations of anti-C5 antibody were sufficient to inhibit >90% of hemolysis in both human (IC_50_ = 2.1 to 3.1 nM) and monkey serum (IC_50_ = 8.2 to 28 nM) (Table 1, S1A and S1B Fig). Effects of anti-C5 antibodies on AP-mediated hemolysis were assessed using rabbit blood cells. Anti-C5 antibodies inhibited AP-mediated hemolysis in human serum with IC_50_ values ranging from 13 to 26 nM, but complete block of AP-mediated hemolysis was not observed for any of the antibodies tested (Table 1, S1C Fig). Using monkey serum, base-line hemolysis was lower (50–70%) than for human serum (93–100%), but anti-C5 antibodies blocked AP-mediated hemolysis with similar potencies (IC_50_ = 7.0 to 9.1 nM) (Table 1, S1D Fig). Collectively, these experiments identified a group of anti-C5 antibodies that bind both human and monkey C5 with high affinity and block complement-induced hemolysis in both normal human and cynomolgus monkey serum.

### REGN3918 has prolonged PK/PD properties in *C5*^*hu/hu*^ mice

Humanized C5 mice (*C5*^*hu/hu*^) were generated to provide an animal model for evaluating the pharmacokinetic (PK) and pharmacodynamic (PD) properties of anti-C5 antibodies in the presence of target. The mouse *C5* gene was humanized by introducing genomic human C5 sequence, including exons 2–41, partial intron 1, the 3’UTR, and approximately 1.5 kb downstream sequence of the human C5 gene, into the corresponding region in mouse *C5* (S2A Fig). The resulting locus encodes secreted human C5 protein and a portion of the murine N-terminal signal sequence. Because C5 expression levels is known to differ between male and female mice, hC5 levels in *C5*^*hu/hu*^ mouse serum were measured by ELISA in two separate cohorts of male and female mice. Human C5 concentrations in *C5*^*hu/hu*^ mouse serum were 55.4 ± 1.7 μg/mL (n = 47) in males and 24.7 ± 0.6 μg/mL (n = 49) in females (S2B Fig), which are modestly lower than the levels that have been reported in humans and for other mouse strains (~80–90 μg/mL, respectively) [13].

**Fig 1:**
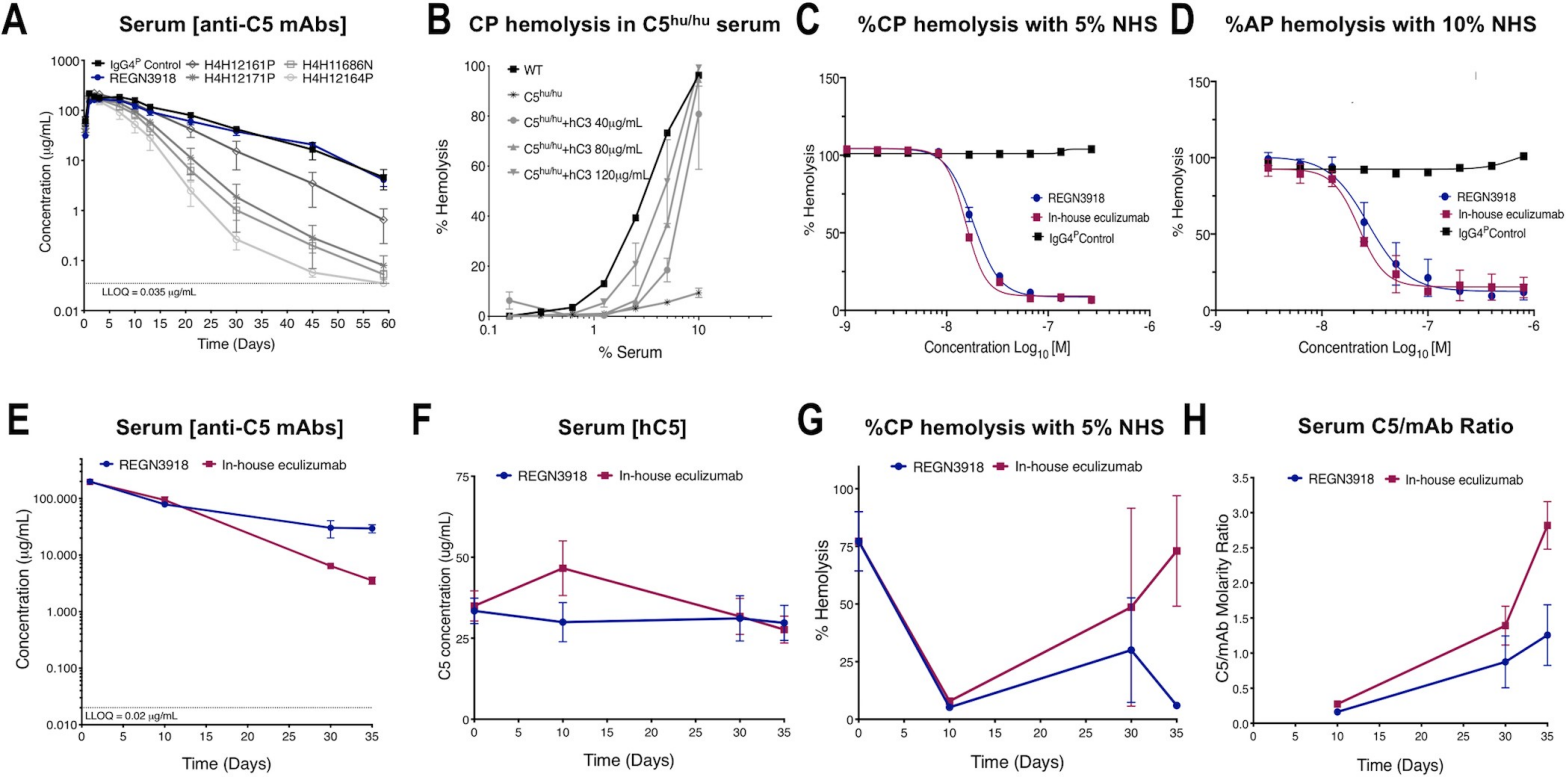
Pharmacokinetic and *ex vivo* pharmacodynamic properties of anti-C5 antibodies in *C5*^*hu/hu*^ mice. (A) Serum concentrations of antibodies were measured by ELISA at 6 hours, 1, 2, 3, 7, 10, 13, 21, 30, 45 and 59 days post dosing in *C5*^*hu/hu*^ mice administered a single 15 mg/kg subcutaneous dose of anti-C5 mAbs or IgG4^P^ control antibody (n = 5). REGN3918 is shown in blue. (B) Supplementing serum from *C5*^*hu/hu*^ mouse with recombinant hC3 (40–120 μg/mL) restores CP-mediated hemolysis to similar levels observed in serum from WT mice. In all cases, hemolytic activity decreased in a dose-dependent manner relative to the amount of serum used in the assay. Dose response curves from one experiment performed in triplicate, with the exception of wild-type (n = 1), at each serum concentration. Experiment was repeated with multiple sera from different donors (n > 3). (C) SRBCs, sensitized with hemolysin in GVB^++^, were incubated for 60 minutes at 37°C with normal human serum, resulting in CP-mediated hemolysis. Pre-incubation of serum with increasing concentrations of REGN3918 or in-house eculizumab blocked hemolysis in a dose-dependent manner. (D) RbRBCs in GVB^++^-Mg^2+^/EGTA were incubated for 15 minutes with normal human serum (NHS), resulting in AP-mediated hemolysis. Pre-incubation of serum with increasing concentrations of REGN3918 or in-house eculizumab blocked hemolysis in a dose-dependent manner. For hemolysis assays (C, D), representative experiments are shown that were performed in duplicate at each mAb concentration; each experiment was repeated with multiple sera from different donors (n > 3). (E) Antibody serum concentrations were measured by Gyros from mice at 1, 10, 30 and 35 days post dose in *C5*^*hu/hu*^ mice administered a single 15 mg/kg subcutaneous dose of anti-REGN3918 (blue) or in-house eculizumab (red), n = 4 (E-F). (F) The hC5 serum concentrations were measured and plotted for mice terminally bled at pre-dose and 10, 30 and 35 days post dosing in *C5*^*hu/hu*^ mice administered a single 15 mg/kg subcutaneous dose of anti-REGN3918 (blue) or in-house eculizumab (red). (G) The percent of CP-mediated hemolysis was determined *ex vivo* using serum from mice terminally bled at predose and 10, 30 and 35 days after administration of REGN3918 (blue) or in-house eculizumab (red). (H) Serum concentrations of hC5 and hIgG were used to calculate the serum C5/mAb ratio in *C5*^*hu/hu*^ mice dosed with anti-REGN3918 (blue) or in-house eculizumab (red). Data is plotted as mean ±SEM (A-F).

*C5*^*hu/hu*^ mice (n = 3–5 for each group) were used to screen the PK properties of high affinity anti-C5 antibodies *in vivo*. Mice received a single 15 mg/kg SC dose, and blood was collected at pre-specified post-injection time-points and serum monoclonal antibody (mAb) concentrations were measured. Surprisingly, the different anti-C5 antibodies exhibited remarkably variable serum half-lives in C5 humanized mice, ranging from 1 to 11 days (Fig 1A, S2C Fig), with REGN3918 having the longest half-life and highest exposure of the antibodies examined. The PK profile of REGN3918 was similar to that of the IgG4^P^ control antibody (T_1/2_ ≈ 10d). To determine if the difference in PK for these anti-C5 antibodies was due to differences in target-mediated clearance or intrinsic clearance properties of the antibodies, a follow-up PK study was performed in wild-type (WT) C57BL/6 mice. Mice were dosed SC with 15 mg/kg of an anti-C5 or IgG4^P^ control antibody (n = 4) and blood was collected at pre-specified post-injection time-points to measure serum mAb concentrations. All six anti-C5 antibodies displayed similar peak concentrations (C_max_ = 152–202 μg/mL) and half-life (T_1/2_ ≈ 12–17 days) values that were comparable to that of the IgG4^P^ control (C_max_ = 177 μg/mL; T_1/2_ ≈ 13 days) (S2D Fig). None of the anti-C5 antibodies tested bind murine C5. Thus, these results demonstrate that in the absence of target (C5) this group of anti-C5 antibodies share similar pharmacokinetics.

These results provided rationale for further evaluating REGN3918 as a potential lead therapeutic molecule. Consequently, additional studies were conducted to better characterize REGN3918 and compare it with an in-house produced version eculizumab. In-house eculizumab was produced according to the published sequence of commercial eculizumab and was found to bind recombinant C5 with similarly high affinity (KD = 301 and 248 pM for in-house and commercial eculizumab, respectively) at 25°C, which was comparable to the binding of REGN3918 to C5 (189 pM) using SPR-Biacore technology (Table 2). In *ex vivo* hemolysis assays, low nanomolar concentrations of REGN3918 or in-house eculizumab were sufficient to block CP (IC_50_ = 18.3 and 15.4 nM, respectively) and AP (IC_50_ = 27.4 and 22.3 nM, respectively) complement-mediated hemolysis using human serum, whereas an IgG4^P^ control antibody had no effect (Fig 1C and 1D, S2E Fig).

**Table 2:** Comparison of kinetic properties for REGN3918 and in-house eculizumab binding to human and cynomolgus monkey C5, C5 variants and C5a/6 complexes at 25°C, pH7.4.

Anti-C5 antibody	Binding to Human C5		Binding to Monkey C5		Binding to Human R885H C5		Binding to Human R885C C5		Binding to Human C5b,6	
	KD [M]	T_1/2_ (min)	KD [M]	T_1/2_ (min)	KD [M]	T_1/2_ (min)	KD [M]	T_1/2_ (min)	KD [M]	T_1/2_ (min)
REGN3918	1.89E-10	61.9	2.73E-09	4.1	4.22E-10	42.6	4.65E-10	43.0	1.37E-10	109.3
In-house eculizumab	3.01E-10	103.5	NB	NB	NB	NB	NB	NB	1.49E-10	294

Abbreviations: KD = dissociation constant; NB = No binding; T_1/2_ = half-life

To better characterize the PK/PD properties of REGN3918, *C5*^*hu/hu*^ mice were administered a single 15 mg/kg SC dose of REGN3918 or in-house eculizumab (n = 4) and blood was collected at pre-specified times to measure serum mAb and C5 concentrations. Again, REGN3918 showed prolonged PK with a half-life of approximately 13 days, which was extended compared to the half-life of in-house eculizumab (T_1/2_ ≈ 6d) (Fig 1E). The findings indicate that, despite its high C5 binding affinity, REGN3918 undergoes limited target-mediated clearance in this humanized mouse model.

The ability of dosed anti-C5 antibodies to block complement-mediated hemolysis was evaluated using the serum of dosed mice in an *ex vivo* CP hemolysis assays. As shown in Fig 1B, compared to serum from WT mice (96% hemolysis) the serum from *C5*^*hu/hu*^ mice induced only 11.1% hemolysis in the CP assay, indicating that the complement pathway is compromised in *C5*^*hu/hu*^ mice. However, supplementing *C5*^*hu/hu*^ mouse serum with 40, 80, or 120 μg/mL human C3 (hC3) fully restored hemolytic activity (102.8%, 94.6%, and 106.6%, respectively). In comparison, supplementing serum from *C5*^*-/-*^ mice with hC3, up to 500 μg/mL, failed to fully restore hemolysis (30%), indicating that hC3 specifically restores hemolysis through reconstitution of a functional complement pathway in *C5*^*hu/hu*^ mouse serum. Consistent with the prolonged half-life of REGN3918, hemolysis was significantly blocked to <10% of baseline over the duration of the 35-day study (Fig 1G). In the REGN3918 group that was terminally bled on day 30, one of the mice had elevated hC5 levels (75 μg/ml) compared to the other two (4 and 10 μg/ml), which resulted in the elevated hemolysis observed at this time point compared to d35. Because these assays were performed using terminal bleeds and mice were not serially bled throughout the study, we were unable to determine whether this was an outlier that may have been improperly dosed. In mice dosed with in-house eculizumab, hemolysis was effectively blocked to <10% 10 days after administration; however, CP-mediated hemolysis returned to baseline levels of 75% hemolysis by day 35 (Fig 1G).

Serum C5 levels were stable over a period of 35 days in mice dosed with REGN3918, whereas in-house eculizumab induced a ~1.5-fold increase over baseline in serum C5 at day 10 (Fig 1F). Interestingly, inhibition of CP hemolysis was found to correlate with the molar ratio of mAb to C5 in serum (Fig 1H). In serum with a molar C5:mAb ratio below two, CP-mediated hemolysis was blocked in groups that received either REGN3918 or in-house eculizumab, which is consistent with previous reports for eculizumab [14]. This included the post-dosing day 35 time point in *C5*^*hu/hu*^ mice administered REGN3918, which had a C5:mAb ratio of 1.3. In contrast, in serum collected 35 days after administration of in-house eculizumab, the molar C5:mAb ratio was 2.8 and the level of CP-mediated hemolysis was comparable to baseline.

### REGN3918 has prolonged *in vivo* half-life and *ex vivo* PD effects in cynomolgus monkeys

**Fig 2:**
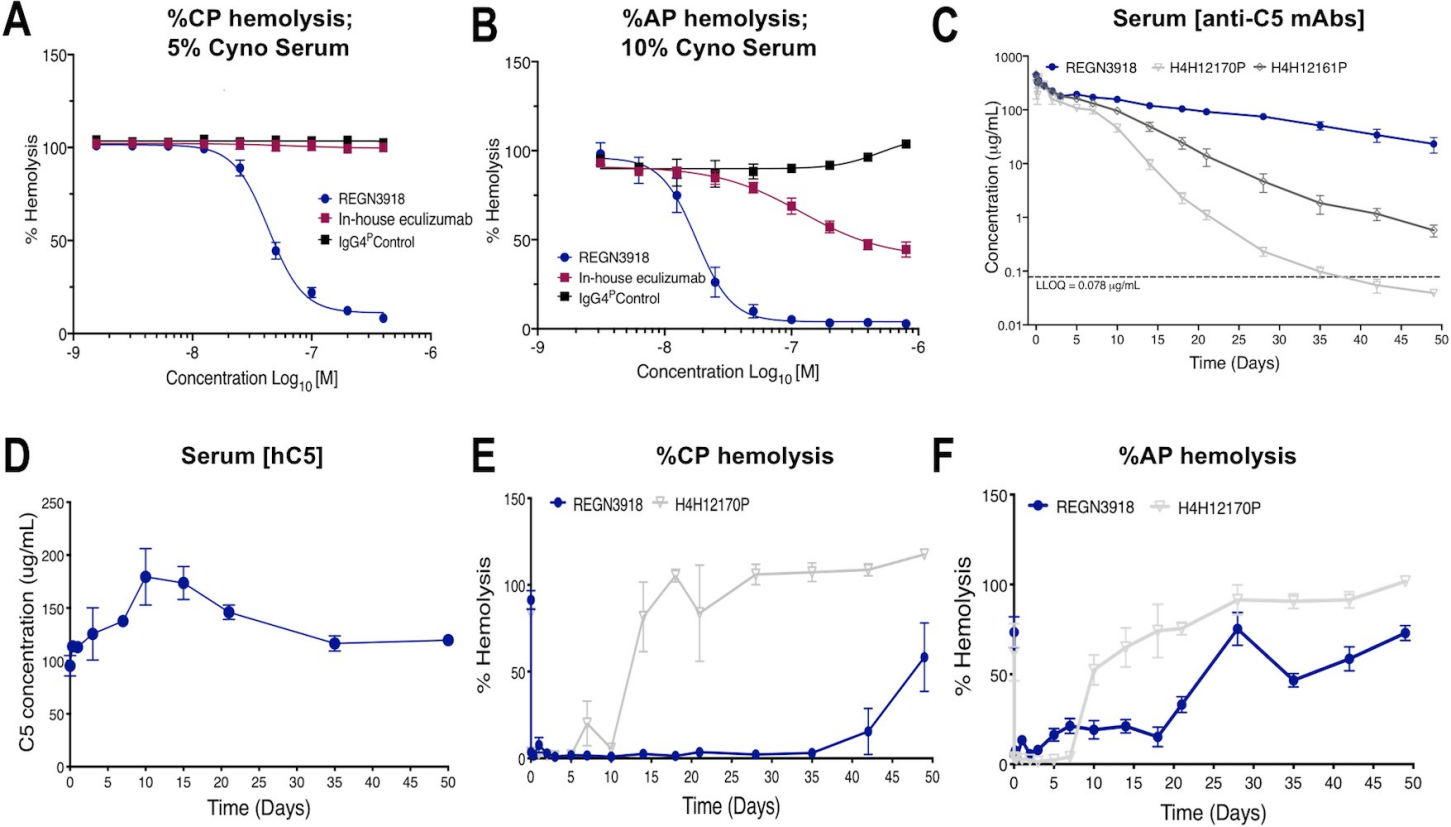
Characterization of the antihemolytic activity of anti-C5 antibodies in monkey serum. (A) SRBCs, sensitized with hemolysin in GVB^++^, were incubated for 60 minutes at 37°C with 5% cynomolgus monkey serum, resulting in CP-mediated hemolysis. Pre-incubation of serum with increasing concentrations of REGN3918 blocked hemolysis in a dose-dependent manner. In-house eculizumab had no significant effect on hemolysis. (B) RbRBCs in GVB^++^-Mg^2+^/EGTA were incubated for 15 minutes with 10% cynomolgus monkey serum, resulting in AP-mediated hemolysis. Pre-incubation of serum with increasing concentrations of REGN3918, but not in-house eculizumab, fully blocked hemolysis in a dose-dependent manner. For hemolysis assays (A, B), representative experiments are shown that was performed in duplicate at each mAb concentration; each experiment was repeated with multiple sera from different donors (n > 3). (C) The serum concentrations of REGN3918 (blue), H4H12161P, and H4H12170P antibodies were measured in cynomolgus monkeys at 5 minutes, 4 and 8 hours, and 1, 2, 3, 5, 7, 10, 14, 18, 21, 28, 35, 42, and 49 days after administration of a single 15 mg/kg intravenous dose of each antibody (n = 4 for C-F). (D) hC5 serum concentrations were measured at predose, 8 hours and 1, 3, 7, 10, 15, 21, 35 and 50 days post dose in cynomolgus monkeys administered a single 15 mg/kg intravenous dose of anti-REGN3918. (E-F) Serum from monkeys dosed with REGN3918 (blue) or H4H12170P were collected at 5 minutes, 4 and 8 hours, and 1, 2, 3, 5, 7, 10, 14, 18, 21, 28, 35, 42, and 49 days and used to examine the percent of CP- (E) and AP-mediated (F) hemolysis. Data is plotted as mean ±SEM.

REGN3918 and other VelocImmune anti-C5 antibodies were found to bind monkey C5 with high affinity (Tables 1 and 2), which provided a strong rationale for further evaluating these antibodies in primates. In-house eculizumab, on the other hand, did not bind monkey C5 under similar conditions, consistent with published results [13,14]. Similarly, *ex vivo* hemolysis assays demonstrated that REGN3918 blocked both CP and AP mediated hemolysis in monkey serum (IC_50_ = 43.4 and 17.8 nM, respectively), whereas in-house eculizumab failed to significantly block hemolysis in either the CP or AP assay using monkey serum (Fig 2A and 2B, S2F Fig). For these reasons, in-house eculizumab was not tested in monkeys *in vivo*.

Evaluation of the PK/PD properties for anti-C5 antibodies in cynomolgus monkeys was performed by intravenous dosing (15 mg/kg) of REGN3918, H4H12161P, or H4H12170P, three anti-C5 antibodies that exhibited a range of fast to slow clearance in the humanized C5 mouse model. Consistent with PK profiles observed in *C5*^*hu/hu*^ mice, REGN3918 displayed the most prolonged half-life in monkeys (T_1/2_ ≈ 14d), whereas the half-life for H4H12161P and H4H12170P were approximately 5 and 4 days, respectively (Fig 2C). Similar C_max_ values were measured for all three antibodies. As a result, monkeys administered REGN3918 had a greater exposure to drug compared to those that received H4H12161P and H4H12170P. Indeed, *ex vivo* hemolysis assays demonstrated that REGN3918 blocks both CP- and AP-mediated hemolysis activity for up to 35 days post-dosing, whereas the H4H12170P treated group failed to inhibit CP- and AP-hemolytic activity beyond 10 and 20 days, respectively (Fig 2E and 2F). In both mouse and monkey, REGN3918 displayed a prolonged PK/PD compared to other anti-C5 antibodies that bind C5 with similar affinity, however the mechanism underlying these favorable properties remain unclear. One difference between the monkey and mouse models was the transient rise in serum C5 levels that was observed in monkeys after administration of REGN3918 (Fig 2D). It is unclear whether the reduced affinity of REGN3918 for monkey C5 and/or other factors contribute to this difference.

### REGN3918 and in-house eculizumab bind distinct regions of C5

To determine whether REGN3918 and eculizumab bind different sites on C5, cross-competition binding assays were performed using real-time, label-free bio-layer interferometry. In these assays, C5 complexed with REGN3918 did not block binding of in-house eculizumab (S3A Fig). Further, in-house eculizumab bound to C5 did not inhibit binding of REGN3918, indicating that the antibodies do not compete for binding to C5.

**Fig 3:**
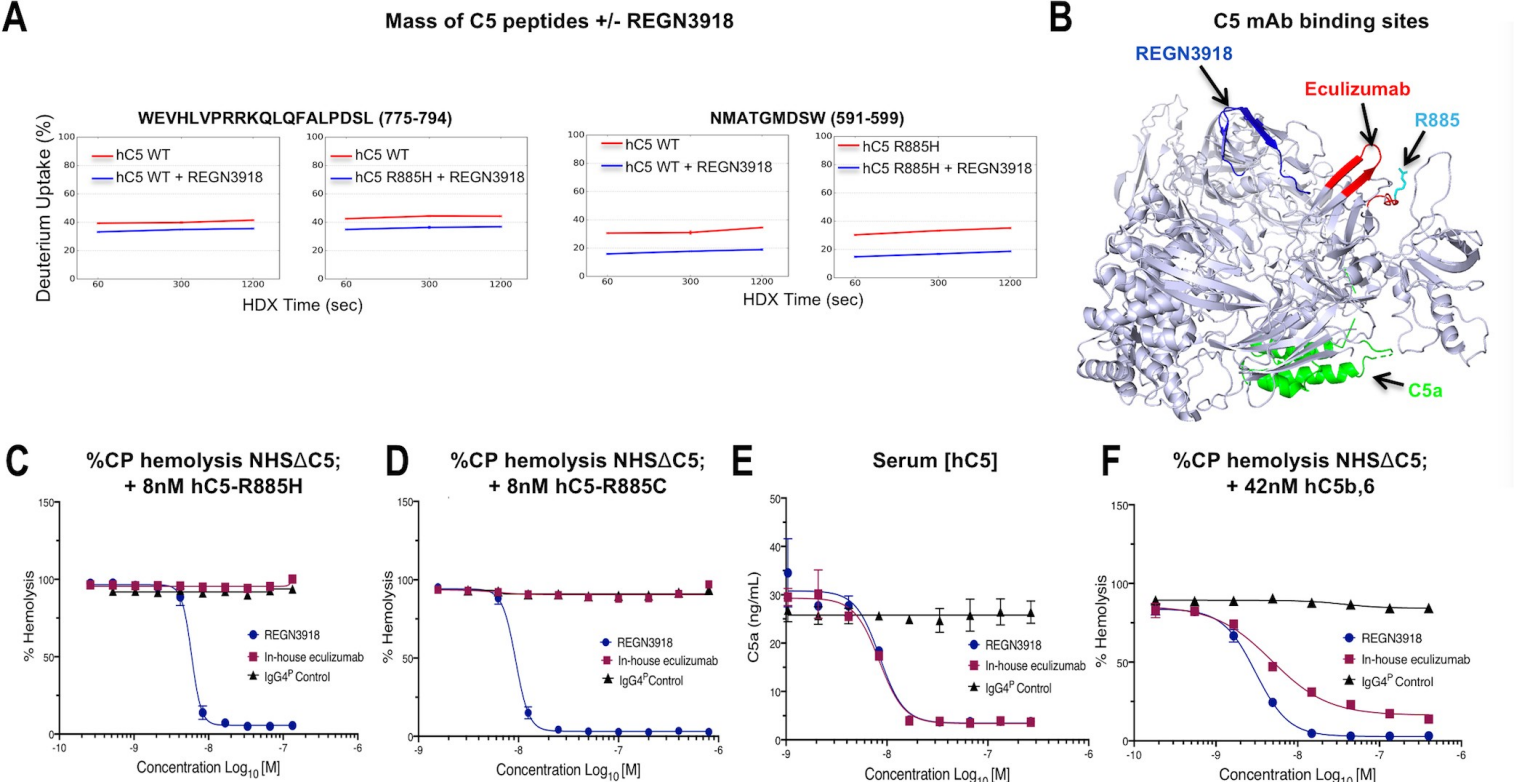
REGN3918 blocks complement-mediated hemolysis mediated by C5 genetic variants and preformed C5b/C6 complexes. (A) Hydrogen-deuterium exchange (HDX) experiments revealed that peptides corresponding to amino acids 591–599 (NMATGMDSW) and 775–794 (WEVHLVPRRKQLQFALPDSL) in C5 showed a slower rate of deuteration uptake when REGN3918 was bound to either the WT C5 or the C5R885H variant compared to unbound C5. Representative result of a HDX experiment performed in duplicate is shown. (B) 3-D cartoon model depicting the crystal structure of C5. The portion of C5 that comprises C5a following cleavage by C5 convertase is depicted in green and amino acid R885 is shown in cyan. The regions of C5 that are involved in binding REGN3918 and eculizumab are shown in blue and red, respectively. (C-D) Normal human serum was immunodepleted of C5, supplemented with 80–90 μg/mL hC3 as well as either 8 nM recombinant C5^R885H^ (C) or 31.6 nM recombinant C5^R885C^ (D) and used in CP-mediated hemolysis assay to determine the percent block of hemolysis over a dose of antibody concentration for REGN3918 (blue), in-house eculizumab (red) and IgG4^P^ control (black). Pre-incubation of serum with increasing concentrations of REGN3918, but not in-house eculizumab and IgG4^P^ control, fully blocked hemolysis in a dose-dependent manner. (E) Concentration of C5a in normal human serum 60 min after inducing CP-mediated hemolysis in the presence of different concentrations of REGN3918 (blue), in-house eculizumab (red) and IgG4^P^ control (black). Both REGN3918 and in-house eculizumab, but not IgG4^P^ control, prevented generation of C5a in a dose-dependent manner. (F) The percent block of CP-mediated hemolysis was determined over a range of antibody concentrations for REGN3918 (blue), in-house eculizumab (red) and IgG4^P^ control (black) in C5 depleted normal human serum supplemented with 80–90 μg/mL hC3 and 42 nM of precomplexed, recombinant C5b/6. Data is plotted as mean ±SD. Representative experiments (C-F) are shown that were performed in duplicate at each mAb concentration; each experiment was repeated with multiple sera from different donors (n > 3).

The interaction of REGN3918 with C5 was examined using hydrogen-deuterium exchange mass spectrometry (HDX-MS). Peptides corresponding to amino acids 591–599 (NMATGMDSW) and 775–794 (WEVHLVPRRKQLQFALPDSL) of C5 showed a slower rate of deuteration when REGN3918 was bound (Fig 3A), indicating these regions of C5 participate in binding REGN3918. This site is distinct from the reported eculizumab epitope [15], which includes C5-R885 (Fig 3B). Patients with C5 variants at this position, C5^R885H^ or C5^R885C^, do not benefit from eculizumab treatment as these amino acid substitutions disrupt binding of eculizumab to C5 (Table 2). However, REGN3918 bound to both of these variants with high affinity (422 and 465 pM for C5^R885H^ or C5^R885C^, respectively) (Table 2). Furthermore, REGN3918 showed identical HDX profiles when tested with C5^R885^ and the C5^R885H^ variant (Fig 3A). Fig 3B illustrates the locations on C5 of the peptides that were protected by REGN3918 in the HDX-MS studies (blue), the C5a cleavage product (green), and R885 (cyan).

### REGN3918 blocks CP-mediated hemolysis in human serum containing rare human C5 genetic variants and preformed C5b/6 complexes

To examine the potential for REGN3918 to block hemolysis in patients carrying rare C5 genetic variants, *ex vivo* CP-mediated hemolysis assays were performed using C5-depleted normal human serum supplemented with either recombinant C5^R885H^ or C5^R885C^. REGN3918 blocked hemolysis in a dose-dependent manner in C5-depleted normal human serum containing either recombinant C5^R885H^ or C5^R885C^ with IC_50_ values of 5.9 and 9.35 nM, respectively (Fig 3C and 3D). In contrast, an IgG4^P^ control antibody and in-house produced eculizumab failed to block CP-mediated hemolysis of these C5 variants under identical assay conditions, suggesting that REGN3918 could provide a treatment option to a broader patient population than eculizumab.

Complement mediated hemolysis requires cleavage of C5 into the split products C5a and C5b, which leads to the assembly of MAC via sequential recruitment of complement components by C5b. To confirm the expected mechanism of action of REGN3918, C5a levels were monitored during *ex vivo* CP-mediated hemolysis to assess C5 cleavage in the presence of REGN3918, in-house eculizumab or an IgG4^P^ control antibody. REGN3918 and in-house eculizumab effectively blocked generation of C5a with similar potency (IC_50_ = 8.5 nM and 8.4 nM, respectively), whereas an IgG4^P^ control antibody had no significant effect on C5a levels (Fig 3E). These results indicate that REGN3918 and in-house eculizumab block terminal complement pathway activation by preventing cleavage of C5 into C5a and C5b.

SPR-Biacore binding experiments demonstrate that REGN3918 and in-house eculizumab also bind to C5b/6 complexes with high affinity (137 and 149 pM, respectively) (Table 2), suggesting that, in addition to inhibiting cleavage of C5, REGN3918 may also block further downstream in the complement pathway. To determine whether REGN3918 may also block activity of C5b/6 complexes, a CP hemolysis assay was performed using C5-depleted normal human serum supplemented with precomplexed, recombinant C5b/6 (42 nM). REGN3918 effectively blocked CP-mediated hemolysis in serum containing C5b/6 protein complexes with an IC_50_ of 3.07 nM (Fig 3F), whereas in-house produced eculizumab incompletely blocked hemolysis (69%) with an IC_50_ of 4.8 nM. In contrast, an IgG4^P^ control antibody showed no efficacy in blocking CP-mediated hemolysis. Thus, REGN3918 can block terminal complement pathway activation by preventing C5 cleavage as well as by blocking at the level of C5b/6 complexes.

### REGN3918, In-house eculizumab and C5 complexes predominantly contain 1 to 2 molecules of C5

REGN3918 has the potential to be a viable therapeutic option for patients that carry rare genetic variants of C5 and may also provide an alternative for patients currently treated with eculizumab. However, combining antibodies that bind unique epitopes on a soluble antigen has the potential to generate higher order protein complexes, which can elicit type III hypersensitivity reactions similar to serum sickness. Such conditions are likely to be self-limiting and the size of the complexes will be influenced by the molar ratio of the antibodies and antigen, with the largest complexes generally forming when the components are at or near equimolar amounts. Here, we examined the size of complexes formed at 5:1:1 molar ratios of REGN3918:in-house eculizumab:C5 by asymmetric flow field flow fractionation using multi-angle laser light scattering detection (A4F-MALLS). This molar ratio was chosen based on the serum concentrations that would be expected *in vivo* at the time of the initial dose switch in the clinic.

Representative fractograms generated following A4F-MALLS analysis of the in-house eculizumab/C5/REGN3918 mixture and each of the individual components are overlaid in S3B Fig. A major peak (Peak 1; ~66% total peak area, Table 3) representing free REGN3918 was detected in the simulated mixture (orange trace), since the concentration of REGN3918 is likely in vast molar excess to both C5 and in-house eculizumab. Several additional minor peaks (Peaks 2–4) corresponding to heteromeric complexes of in-house eculizumab, C5, and REGN3918 were also detected in this sample, confirming that both in-house eculizumab and REGN3918 can engage the same molecule of C5 to form extended antibody-antigen lattices. However, the majority of these complexes fractionate into two discrete peaks (Peaks 2 and 3; ~22% total peak area) with calculated average molar masses of ~499 kDa and ~841 kDa. Based on the calculated molar masses of the individual components, peaks 2 and 3 likely represent 2:1 and 3:2 mAb:C5 heteromeric complexes, respectively. A broad, poorly-resolved peak (Peak 4) that likely corresponds to a heterogenous distribution of higher order complexes (~1200–2100 kDa) was also detected, but only represented ~12% of the total peak area. Taken together, this data suggests that although in-house eculizumab and REGN3918 can form heteromeric complexes with C5, the formation of very large, heterogeneous, and potentially immunogenic complexes is likely minimal when each component is present at concentrations expected *in vivo* at the time of the initial dose switch. Furthermore, the formation of these very large complexes is likely transient and should steadily decline as eculizumab is cleared from circulation and/or with additional doses of REGN3918.

**Table 3:** Size distribution of heteromeric complexes formed by REGN3918, in-house eculizumab and C5.

[REGN3918] μM	[In-house eculizumab μM	[hC5] μM	Approximate Molar Ratio	Peak 1		Peak 2		Peak 3		Peak 4	
				Free mAb / hC5		[mAb]_2_: [hC5]_1_ Complex		[mAb]_3_: [hC5]_2_ Complex		Higher Order Heteromeric Complexes	
				Mean MW (kDa)	Mean Peak Area (%)	Mean MW (kDa)	Mean Peak Area (%)	Mean MW (kDa)	Mean Peak Area (%)	Mean MW (kDa)	Mean Peak Area (%)
6.7	0	0	1:0:0	153.9 (0.9)	100 (0.0)	NA	NA	NA	NA	NA	NA
0	6.7	0	0:1:0	151.6 (0.0)	100 (0.0)	NA	NA	NA	NA	NA	NA
0	0	5.3	0:0:1	191.8 (0.5)	100 (0.0)	NA	NA	NA	NA	NA	NA
4.3	0.7	0.8	5:1:1	153.5 (0.5)	64.6 (0.6)	498.8 (3.7)	12.6 (0.7)	840.9 (3.2)	9.1 (0.6)	~1200–2100	13.8 (0.6)

NA = Not applicable

^a^ Weight-averaged molar mass (kDa) is reported with standard deviation in parentheses. Reported values were obtained from duplicate injections of a single sample preparation.

^b^ Peak area percentages were calculated from the UV-vis detector for each sample injected onto the column. Each sample was injected in duplicate and the average peak area percentages are reported

### Switching from in-house eculizumab to REGN3918 results in normalization of C5 concentrations in *C5*^*hu/hu*^ mice and sustained suppression of hemolytic activity *ex vivo*

**Fig 4:**
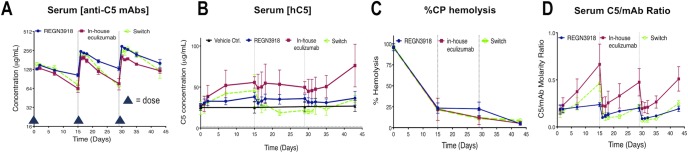
Dose switching from in-house eculizumab to REGN3918 results in normalization of serum C5 concentrations and maintained suppression of hemolytic activity. (A) Total hIgG concentration was measured by Gyros in serum collected from *C5*^*hu/hu*^ mice administered 3 doses of REGN3918 alone (blue), 3 doses of in-house eculizumab alone (red), or 1 dose of in-house eculizumab followed by 2 doses of REGN3918 (switch, green) (≥14 mice/group). Arrowheads on y-axis and vertical grey dashed lines indicate times of dosing. (B) Total C5 serum concentrations in *C5*^*hu/hu*^ mice administered REGN3918 alone (blue), in-house eculizumab alone (red), or switched from in-house eculizumab to REGN3918 (switch, green) were measured from mice bled over the duration of the study. (C) Serum collected from terminally bled *C5*^*hu/hu*^ mice administered with REGN3918 alone (blue), in-house eculizumab alone (red), or in-house eculizumab/REGN3918 switched (green) were supplemented with hC3 and the percent of CP-mediated hemolysis using *ex vivo* assays was assessed. (D) Serum concentrations of total C5 and hIgG were used to calculate the ratio of C5:mAb at the indicated times. Data is plotted as mean ±SEM.

To assess the effects of switching treatments from in-house eculizumab to REGN3918, three groups of *C5*^*hu/hu*^ mice (≥14 mice/group) were administered three doses of 15 mg/kg of REGN3918 or in-house eculizumab on days 0, 15 and 29. One group received REGN3918 only for all three doses, while a second group received in-house eculizumab only. The third group, the ‘switch group’, received in-house eculizumab on day 0 and was then switched to REGN3918 on days 15 and 29. Cage-side observations and routine veterinary staff wellness checks showed mice to be healthy with all animals surviving until their scheduled date of termination. Over the duration of the study, blood was sequentially collected at multiple points pre- and post-dosing. C_max_ values for REGN3918 and in-house eculizumab were comparable following the first dose (151 and 144 μg/mL, respectively); however, REGN3918 alone demonstrated slower clearance (CL) compared to in-house eculizumab alone, resulting in modestly higher serum concentrations for REGN3918 (Fig 4A, Table 4). Following the switch from in-house eculizumab to REGN3918, the concentration-versus time profile of total hIgG initially resembled the PK profile of REGN3918 during the second post-switch dosing interval (Fig 4A, Table 4). These results suggest that there is only a modest effect on the total IgG concentrations in serum upon switching from in-house eculizumab to REGN3918 relative to the concentrations observed with either mAb administered as a single agent.

**Table 4:** PK/PD parameters of antibodies in *C5*^*hu/hu*^ mice dosed with in-house eculizumab alone, REGN3918 alone or switched from in-house eculizumab to REGN3918.

Parameter	Units	Day 0–15			Day 16–29			Day 30–43		
		REGN3918	In-house eculizumab	Switch	REGN3918	In-house eculizumab	Switch	REGN3918	In-house eculizumab	Switch
C_max_	μg/mL	151 ± 18	144 ± 15	162 ± 10	253 ± 12	200 ± 27	238 ± 4.2	298 ± 22	203 ± 20	291 ± 14
AUC_Last_	d•μg/mL	1800 ± 230	1560 ± 310	1770 ± 160	2460 ± 240	1750 ± 380	1940 ± 71	2960 ± 450	2080 ± 74	2860 ± 540
CL	mL/d/kg	3.3 ± 1	6.4 ± 5	5.0 ± 2	3.0 ± 1	6.0 ± 3	5.7 ± 0.2	2.5 ± 1	2.8 ± 0.8	3.1 ± 2
CP Hemolysis	%	23.4 ± 6.1	33.2 ± 16.0	ND	22.5 ± 8.0	12.0 ± 8.4	11.4 ± 0	4.8 ± 0.23	5.2 ± 2.3	8.3 ± 2.2
AP Hemolysis	%	6.9 ± 0.4	10.4 ± 5.1	ND	8.2 ± 3.8	6.8 ± 1.7	4.7 ± 0	5.6 ± 0.5	9.3 ± 3.7	7.2 ± 0.5

C_max_ = Maximum serum concentration; AUC_Last_ = Area under the concentration versus time curve from time zero to the time of the last measurable concentration; CL = total body clearance of drug from serum; CP = classic pathway; AP = alternative pathway; ND = not determined

Serum C5 concentrations were also monitored. In mice administered REGN3918, serum concentrations of C5 increased to a maximum of 1.4-fold over the duration of the study. In contrast, in-house eculizumab induced higher concentrations of serum C5 after the first, second and third doses (1.9, 2.0 and 2.8-fold increases, respectively) (Fig 4B). As expected, the first dose of in-house eculizumab administered to the ‘switch group’ induced a similar increase in serum C5 concentrations as in animals that received in-house eculizumab alone. After switching treatment to REGN3918 on day 15, serum C5 concentrations in the ‘switch group’ transiently fell below baseline (70%), but returned to levels similar to the group administered REGN3918 alone after the final dose of REGN3918. The accelerated clearance of C5 following dose switching may be consistent with a transient formation of immune complexes containing REGN3918, in-house eculizumab and C5, as demonstrated by A4F-MALLS studies.

The effects of switching treatments from in-house eculizumab to REGN3918 on the efficacy of blocking complement-mediated hemolysis were also assessed. Sera from terminally sacrificed *C5*^*hu/hu*^ mice (n = 5) were collected prior to each new dose as well as 14 days post third dose and used in AP- and CP-mediated hemolysis assays. Hemolysis was effectively blocked to a similar extent in serum collected from all three groups after the first, second and third administration of antibody (Fig 4C, S2G Fig). Blockage of hemolysis was associated with C5:mAb ratios that were maintained below one over the duration of the study (Fig 4D). Collectively, these results indicate that switching treatments from in-house eculizumab to REGN3918 was generally well tolerated and was associated with continued suppression of complement activation *ex vivo*.

## Discussion

Inhibition of C5 is a clinically validated mechanism to treat paroxysmal nocturnal hemoglobinuria (PNH) and atypical hemolytic uremic syndrome (aHUS) which are life-threatening orphan diseases characterized by over-activation of terminal complement activity [11,16]. In this study involving humanized C5 mice, we describe the generation and characterization of a fully humanized anti-C5 monoclonal antibody, pozelimab (REGN3918), that binds to human C5 with high affinity, effectively inhibits C5 induced hemolysis and demonstrates prolonged PK/PD profiles. Further, we demonstrate that *C5*^*hu/hu*^ mice provide an excellent tool for screening anti-C5 antibodies and may be predictive of how anti-C5 antibodies behave in higher organisms.

A major challenge to developing a therapeutic C5 antibody is the lack of an animal model for evaluating antibody PK/PD properties *in vivo*. The anti-human C5 antibodies that bound C5 with high-affinity and blocked hemolysis in normal human serum isolated in our screen did not bind murine C5 or block hemolysis using mouse serum. This precludes the possibility of using wild-type mice to evaluate the PK/PD properties of many potential anti-C5 antibody therapeutics.

In the present study, we leverage humanized C5 mice (*C5*^*hu/hu*^) to evaluate the PK/PD characteristics of anti-C5 antibodies in the presence of C5 engagement and ongoing C5 synthesis *in vivo*. Screening of antibodies in *C5*^*hu/hu*^ mice identified REGN3918 as the lead anti-C5 therapeutic based on its favorable in vitro properties and prolonged PK profile that was similar to the IgG4^P^ control (T_1/2_ ≈ 11–13 and 10 days, respectively), in contrast to other anti-C5 antibodies that were subject to significant target-mediated clearance. Further, *ex vivo* hemolysis assays using the serum from C5 humanized mice were used to measure activity of the classical and alternative complement pathways after antibody dosing, which confirmed that the prolonged half-life of REGN3918 translated into efficient block of C5-mediated hemolysis beyond what was observed for other anti-C5 antibodies.

An alternative strategy for improving the PK/PD properties of an antibody is to engineer antibodies to bind target with pH-dependence [17]. This approach aims to reduce antigen binding at more acidic pH, compared to neutral pH, in order to promote dissociation of the antibody from antigen in the endosome, where FcRn receptors can capture the antibody and recycle it back into the circulation [18]. A common method for engineering pH sensitive antibodies is to introduce histidine residues, which become protonated at lower pH, into the variable domains of antibodies. This strategy was used in the development of ALXN1210, a modified version of eculizumab, that harbors two variable domain histidine mutations and amino acid substitutions in the Fc domain to increase FcRn binding affinity [19]. Though ALXN1210 is reported to have prolonged PK in humans (T_1/2_ ≈ 32 days), it binds C5 with reduced affinity (17-fold reduction) at neutral pH [19], which may necessitate the administration of higher antibody concentrations to sustain suppression of C5 cleavage *in vivo*.

Up-front screening of antibodies with different binding affinities for target at neutral and acidic pH is another effective strategy for isolating antibodies with improved PK/PD properties. This method, in combination with subsequent engineering of variable domain to optimize affinity to C5, pH dependent binding, stability and solubility as well as the introduction of M428L/N434A mutations to enhance FcRn binding, was used to produce the anti-C5 antibody, SKY59, which has a prolonged half-life in cynomolgus monkeys [20].

REGN3918 was selected for clinical development based on the prolonged PK/PD parameters of this antibody in both humanized mouse and monkey models. Many factors can contribute the PK/PD behavior of antibody therapeutics including immunogenicity, charge, glycosylation, and the properties of how an antibody engages its target and Fc receptors. To determine whether pH-dependent target engagement may contribute to the prolonged PK/PD profiles of REGN3918, binding of REGN3918 to C5 at neutral and acidic pH was examined using SPR-Biacore. Both in-house eculizumab and REGN3918 showed some effects of low pH on hC5 binding (t1/2 ratio of binding hC5 at pH7.4/pH6.0 = 5.2 and 3.8, respectively), but the effects were similar and did not help explain their differences in clearance *in vivo*. It has been reported that calcium-dependent binding of antigen can also influence the recycling of antibodies from the endosome ^20^. Therefore, we also investigated the calcium-dependence of binding between REGN3918 and C5 using SPR-Biacore, but found that the C5 binding affinity of REGN3918 was not significantly influenced by the presence or absence of calcium (K_D_ = 589nM -EDTA, 221nM +EDTA at 25°C) and indicating that alternative mechanisms may underlie the prolonged PK of REGN3918 in *C5*^*hu/hu*^ mice and cynomolgus monkeys.

Another factor that can contribute to the improved PK of anti-C5 antibodies that recycle through the endosome is that unbound, internalized C5 is cleared through lysosomal degradation, rather than being cleared as part of a larger antibody-antigen complex [20]. In the latter case, total C5 is expected to accumulate in plasma, which may be consistent with the reported ~100 μg/mL trough concentration of eculizumab, which is sufficient to neutralize ~ 200 μg/mL of C5 (approximately 3-fold greater than the C5 concentration in the plasma of healthy individuals) [21]. In our study, a single dose of 15 mg/kg in-house eculizumab induced a ~2-fold increase of total C5 in humanized mice at day 7. A similar pattern of serum C5 accumulation was observed in monkeys dosed with LFG316 [22]. In contrast, only a moderate increase in total C5 serum concentrations (~1.2- and 1.8-fold in *C5*^*hu/hu*^ mice and monkeys, respectively) was induced by REGN3918. The contribution of other antibody characteristics, such as charge, glycosylation and target engagement to the prolonged PK of REGN3918 remains unknown, but remains an active area of research.

A4F-MALLS analysis demonstrated that REGN3918 and in-house eculizumab do not compete for C5 binding. These data are supported by HDX-MS experiments showing that REGN3918 bound two discontinuous epitopes in C5, aa591-599 and aa775-794, whereas in-house eculizumab bound the R885 containing peptide aa865-897. These data are consistent with X-ray and cryogenic electron microscopy studies showing that R885 is in close proximity to the C5 epitope for eculizumab [15] and help to explain the loss of binding that is observed for in-house eculizumab to rare human C5^R885H^ and C5^R885C^ variants, and the ability of REGN3918 to bind these same variants with high-affinity. The data also provide an understanding for how REGN3918 completely inhibited hemolytic activity in C5 depleted normal human serum exogenously supplemented with C5^R885H^ or C5^R885C^, whereas in-house eculizumab was not able to block hemolysis under the same conditions. Like eculizumab, REGN3918 is also capable of blocking hemolysis mediated by preformed C5b/C6 complexes. The mechanism for this remains unclear, but may result from blocking the recruitment of other MAC components.

Based on the PK/PD profile for REGN3918 in mouse and monkey studies, pozelimab may require less frequent dosing regiments and result in more stable serum C5 levels. Dose switching studies in humanized C5 mice demonstrated that switching treatments from in-house eculizumab to REGN3918 was well tolerated and maintained suppression of complement activity. However, combining antibody therapeutics against a soluble antigen has the potential to generate higher order, immunogenic protein complexes. Using molar ratios of in-house eculizumab:REGN3918:hC5 that would be expected at the time of dose switching, A4F-MALS studies demonstrated in-house eculizumab and REGN3918 can form heteromeric complexes with C5. However, the formation of very large, heterogeneous, and potentially immunogenic complexes was minimal and would likely be transient *in vivo*. These data may support using an excess of pozelimab when dose switching from eculizumab to minimize the potential for inducing serum sickness-like reactions. Recently, pozelimab (REGN3918) successfully completed phase 1 [23] and has advanced to phase 2 in PNH (NCT03946748).

New therapeutic strategies, including anti-sense oligonucleotides (ASO) or RNAi therapeutics, have the potential to transform treatment options for aHUS and PNH patients by achieving long-term knockdown of C5 expression in the liver. Recent clinical studies showed that treatment of PNH patients using RNAi based therapies that targeted C5, controlled disease associated hemolysis [24]. However, a disadvantage of these therapies is that there is a significant lag between the time of drug administration and efficacy and the lack of complete C5 silencing with current agents. Therefore, it may be advantageous to combine these novel treatment strategies with existing therapies, such as anti-C5 antibodies, to control hemolysis between doses and in the event of breakthrough hemolysis. These potential therapeutic options may provide opportunities to improve the care of patients with PNH, aHUS and other serious diseases characterized by overactivation of the complement system.

## Materials and methods

### Generation of REGN3918

REGN3918 is a fully human monoclonal antibody to human complement C5 produced with IgG4^P^ Fc domains. The IgG4 constant domain contains a serine to proline amino acid substitution (S228P, EU numbering) in the hinge region that reconstructs the human IgG1 hinge sequence (CPPC), used to promote stabilization of disulfide bonds between the two heavy chains [25], and therefore is designated IgG4^P^. Briefly, VelocImmune® mice [12] were immunized with human C5 complement protein using standard techniques. Isolation of C5 reactive B-cells was performed using previously described methods [26]. Anti-C5 antibodies were produced in Chinese hamster ovary (CHO) cells after stable transfection with paired expression plasmids containing heavy chain and light chain derived from the same B cell. Antibodies were purified by Protein A affinity chromatography.

### SPR Biacore assays

Using SPR-Biacore, the initial anti-C5 mAbs binding kinetics were screened on Biacore T200 instrument using a dextran-coated (CM4) chip at 37°C. The running buffer was using filtered HBS-EP (10 mM Hepes, 150 mM NaCl, 3.4 mM EDTA, 0.05% polysorbate 20, pH 7.4). A capture sensor surface was prepared by covalently immobilizing goat anti-human Fc antibody (GE Healthcare Life Sciences, Marlborough, MA, USA) to the chip surface using (1¬Ethyl-3-[3-dimethylaminopropyl]carbodiimide hydrochloride)/N-hydroxysuccinimide coupling chemistry. Anti-C5 monoclonal antibodies were captured through their Fc regions by an anti-human Fc-specific antibody immobilized on the sensor chips and were tested for binding to the human C5 (MilliporeSigma Headquarters, Burlington, MA, USA) and recombinant monkey (*Macaca fascicularis)* C5. C5 proteins (with C-terminal mycmychis) were prepared at concentration ranges between 100 to 0.78 nM and individually injected over captured surfaces for 2 minutes followed by an 8 minute dissociation phase. All capture surfaces were regenerated with one 15-second pulse of 10 mM glycine–HCl (pH 1.5, (GE Healthcare, Marlborough, MA)). Kinetic parameters were obtained by globally fitting the data to a 1:1 binding model with mass transport limitation using curve fitting Scrubber software version 2.0c and Biacore T200 Evaluation. The equilibrium dissociation constant (KD) was calculated by dividing the dissociation rate constant (kd) by the association rate constant (ka).

Biacore binding was performed at 25°C to compare the binding affinity of REGN3918 and in-house eculizumab to human wild-type C5 isolated from serum, recombinant human C5 variants (C5_R885H.mmH and C5_R885C.mmH), recombinant cynomolgus monkey C5 (MfC5.mmH),); as well as C5b/6 isolated from serum. Varying concentrations of C5 and C5 split product proteins were injected over sensor surface-captured REGN3918 at 25°C and pH 7.4. Binding signal changes were recorded, and kinetic binding parameters were determined using a 1:1 binding model with mass transport limitation.

### Generation of humanized C5 mice

The mouse C5 locus was humanized using a vector that contained genomic human C5 sequence including exons 2–41, partial intron 1, the 3’UTR, and approximately 1.5 kb downstream sequence of the human C5 gene, followed by a neo cassette flanked by 2 loxP sites to replace the corresponding region of mouse C5 (the mouse sequence replaced downstream of the 3’UTR was roughly 900bp). The neo cassette was subsequently removed. The humanized locus encodes the secreted human C5 protein and a portion of the murine N-terminal signal sequence.

### Cross-competition binding experiments

The ability of in-house eculizumab to compete with REGN3918 for binding to C5 was examined using real-time, label-free bio-layer interferometry on an Octet instrument at pH 7.4 and 25°C. Anti-hFc biosensor tips were immersed in a well containing either in-house eculizumab, REGN3918 or an IgG4^P^ control antibody. Then, free anti-hFc was blocked with non-binding hIgG. Biosensor tips with surface-captured mAbs were immersed in wells containing soluble C5. These pre-complexed mAb-C5 biosensor tips were then immersed in solutions of each mAb as the second antibody binding step. Binding responses (nm) of the second antibodies to the surface-captured mAb-C5 was recorded for each combination. All samples were prepared in HBS-ET containing 0.1 mg/mL BSA. Data were processed and analyzed using Scrubber software version 2.0c.

### Animal studies

All mice were generated in-house at Regeneron and monkeys were provided by and housed at Covance. All mouse and non-human primate studies were conducted under the protocols approved by the appropriate Institutional Animal Care and Use Committees (IACUCs) (Regeneron’s IACUC committee and Covance Laboratories, Inc. IACUC committee) and the animal ethics committee approved all the animal work performed for this study in its entirety. The studies were conducted in compliance with all applicable sections of the Final Rules of the Animal Welfare Act regulations (Code of Federal Regulations, Title 9), the Public Health Service Policy on Humane Care and Use of Laboratory Animals from the Office of Laboratory Animal Welfare, and the Guide for the Care and Use of Laboratory Animals from the National Research Council. IACUC protocol # 169 was used for mouse studies. If a mouse was determined to be in overt pain/distress, or appeared moribund and beyond a point where recovery appears reasonable, the animal was euthanized. Mice were monitored for the following signs of illness, i.e. matted fur, changes in body weight from insufficient food/water intake, little/no movement, dull eyes and body condition scoring. Any and all observations are documented. For euthanasia of mice, overdose of anesthetic (200 mg/kg ketamine, 20mg/kg xylazine) was administered.

All non-human primate studies were conducted at contract research organization (CRO) laboratories fully accredited by the Association for the Assessment and Accreditation of Laboratory Animal Care (AAALAC), in compliance with the Animal Welfare Act, the Guide for the Care and Use of Laboratory Animals, and the Office of Laboratory Animal Welfare under the Regeneron stuy number: REGN2913-PK-15063 (CRO study number 8324374). The study was conducted according to a protocol and procedures approved by an Institutional Animal Care and Use Committee (IACUC). Veterinary care was available throughout the study by an attending Laboratory Animal Veterinarian. In accordance with applicable animal welfare guidelines, any medical treatment necessary to prevent unacceptable pain and suffering, including euthanasia, is the sole responsibility of the attending Veterinarian. Animals were housed in stainless steel cages, individually housed and co-mingled according to laboratory standard operating procedures (SOP). Animal rooms were temperature controlled with 12-hour light/12-hour dark cycles. Animals were provided feed and water (ad libitum), and other appropriate treats including fruits, as well as in-cage enrichment devices in accordance with applicable laboratory SOPs. Animals were monitored on a daily basis via cage side observations for general health and appearance. Upon completion of the in-life portion of the study animals were returned to the laboratory stock colony.

### In vivo PK study in wild-type (WT) mice

Female WT C57BL/6J mice, 10 weeks old (n = 4), received a single SC injection of 15 mg/kg of anti-C5 mAbs or IgG4^P^ control antibody on day 0. Mice were serially bled at 6 hours, 1, 2, 3, 8 and 14 days via tail bleeds. All blood was processed into serum and stored at -80°C until analysis.

### In vivo PK/PD studies in C5^hu/hu^ mice

Male and female *C5*^*hu/hu*^ mice, 20–30 weeks old (n = 3–5), were stratified based on initial C5 concentrations obtained via tail bleed. Mice received a single SC injection of 15 mg/kg of anti-C5 mAbs or IgG4^P^ control antibody on day 0. Mice in a staggered sacrifice study had terminal blood samples taken via cardiac puncture at 0 (predose) 1, 10, 30 and 35 days. Mice in other PK studies were serially bled at 6 hours, 1, 2, 3, 7, 10, 13, 21, 30, 45 or 59 days or 2, 4, 7, 10, 14 or 18 days via tail bleeds with terminal bleeds taken via cardiac puncture. All blood was processed into serum and stored at -80°C until analysis.

### Switch study in C5^hu/hu^ mice

Male and female *C5*^*hu/hu*^ mice, 16 weeks old (≥14 mice/group), were stratified based on initial C5 concentrations obtained via tail bleed. All mice received SC injections of 15 mg/kg of REGN3918 or in-house eculizumab at 0, 15 and 29 days. Mice for PK analysis were serially bled via tail vein at 0 (predose), 1, 2, 7, 15, 16, 17, 18, 22, 29, 30, 31, 32, 35 and 43 days (n = 2–13/timepoint). Bleeds on dosing days were taken prior to dosing. Mice for PD analysis were terminally bled via cardiac puncture on 0, 15, 29 and 43 days. All blood was processed into serum and stored at -80°C until analysis.

PK parameters for mouse studies were determined by non-compartmental analysis using Phoenix WinNonlin software (version 6.3) and an extravascular dosing model. All mouse studies were approved by the Institutional Animal Care and Use Committee (IACUC) at Regeneron Pharmaceuticals.

### Total hIgG concentrations for PK/PD studies

Total antibody concentrations of anti-C5 mAbs or IgG4^P^ control antibody were determined by an immunoassay specific for detecting human IgG using either the GyroLab xPlore^™^ (Gyros) or a sandwich enzyme-linked immunosorbent assay (ELISA). For Gyros, briefly, a biotinylated goat anti-hFcγ polyclonal antibody (Jackson ImmunoResearch Laboratory) was captured onto streptavidin coated beads in order to capture the human IgG present in the sera. After affinity column capture, bound human IgG antibody in samples was detected with Alexa-647 labeled goat anti-hFcγ polyclonal antibody (Jackson ImmunoResearch Laboratory). Fluorescent signal on the column allowed for the detection of bound IgG and response units (RU) were read by the instrument. Sample concentrations were determined by interpolation from a standard curve that was fit using a 5-parameter logistic curve fit using the Gyrolab Evaluator Software. The lower limit of quantitation (LLOQ) for this assay is 0.02 μg/mL. For ELISA, briefly, a goat anti- hFcγ polyclonal antibody (Jackson ImmunoResearch Laboratory) was passively adsorbed to a microtiter plate overnight at 4°C followed by a nonspecific binding block. Serum samples were allowed to bind to plates for one hour at room temperature. After washing, plate captured human IgG antibodies were detected using a horseradish peroxidase (HRP)-conjugated goat anti-hFcγ polyclonal antibody (Jackson ImmunoResearch) followed by addition of the chromogenic HRP-substrate, 3,3',5,5'-tetramethylbenzidine (TMB; BD Pharmingen). TMB was used to detect HRP activity and the resultant OD_450_ was read on a Perkin Elmer Victor X4 Multimode Plate Reader. Sample concentrations were determined by interpolation from a standard curve that was fit using a 4-parameter logistic curve fit using Prism GraphPad (version 6). The LLOQ for this assay is 0.035 μg/mL.

### Total hIgG concentrations for WT mouse PK and switch study

Total antibody concentrations of REGN3918 or in-house Eculizumab were determined by an immunoassay specific for detecting human IgG using the Gyros. Briefly, a biotinylated mouse anti-human IgG1/IgG4 in-house generated monoclonal antibody (mAb) was captured onto streptavidin coated beads in order to capture the human IgG present in the sera. After affinity column capture, bound human IgG antibody in samples was detected with Alexa-647 labeled mouse anti-human *kappa* light chain in-house generated mAb. Fluorescent signal on the column allowed for the detection of bound IgG and response units (RU) were read by the instrument. Sample concentrations were determined by interpolation from a standard curve that was fit using a 4-parameter logistic curve fit using the Gyrolab Evaluator Software. The LLOQ for this assay is 0.2 μg/mL.

### Concentration of total human C5 by ELISA

In the PK/PD studies and the switch study, concentrations of total human C5 in mice prior to dosing with anti-C5 antibodies or IgG4^P^ control was determined by a sandwich ELISA. Briefly, a mouse anti- human C5 in-house generated mAb was passively adsorbed to a microtiter plate overnight at 4°C followed by a nonspecific binding block. Serum samples were allowed to bind to plates for one hour at room temperature. After washing, plate captured C5 was detected using a mouse anti-C5 in-house generated mAb followed by detection with HRP-conjugated goat anti- mouse Fcγ polyclonal antibody (Jackson ImmunoResearch). The chromogenic HRP-substrate, TMB (BD Pharmingen) was used to detect HRP activity and the resultant OD_450_ was read on a Perkin Elmer Victor X4 Multimode Plate Reader. Sample concentrations were determined by interpolation from a standard curve that was fit using a 4-parameter logistic curve fit using Prism GraphPad (version 6). The LLOQ for this assay is 0.02 μg/mL.

### Concentration of total C5 in cynomolgus monkey and C5^hu/hu^ mice by LC-MRM-MS-based assay

In the cynomolgus monkey PK/PD study and the mouse PK/PD and mouse switch studies, concentrations of total human C5 in serum were determined by measuring the concentration of a unique tryptic peptide LQGTLPVEAR (LQG) in the C5 protein as a surrogate for C5. Theoretically, this method could also detect C5b, the cleaved product of C5. However, due to the instability of free C5b, the concentration of C5b in serum are generally low with the majority of C5b being bound to cell surfaces as MAC complexes ^27,28^. Therefore, the measured amounts are likely to include only negligible amount, if any, of C5b fragment.

For analysis of total C5 in monkey serum, C5 (CompTech, Tyler, TX) was used as the reference standard for the calibration; and a peptide bearing a C-terminal stable isotope-labeled (SIL) arginine residue was used as the internal standard (LQGTLPVEAR-^13^C_6_^15^N_4_). Calibration standards were prepared at concentrations ranging from 3.9 to 250 μg/mL in blank monkey serum (BioIVT, Hicksville, NY). Calibration standards, blanks, and study samples (10 μL each) were dried in Speed-Vac and then denatured in 100 μL of 8M urea/20mM Tris(2-carboxyethyl) phosphine (TCEP) buffer at 37°C for 1 hour. After 10 μL of 25 nM internal standard was added, samples were then alkylated with 10 mM of 2-iodoacetamide at room temperature for 30 minutes before diluted using 50 mM ammonium bicarbonate to a final volume of 500 μL. The samples were then digested by trypsin (1:20 w/w) overnight at 37°C. LQG derived from C5 was detected and quantified by LC-MRM-MS (*m/z* 542.3→571.3) using a Waters Xevo TQ-S coupled with ACQUITY UPLC system. 10 μL of digestion mixture was directly injected onto a Waters BEH Peptide C_18_ Column (130 Å, 1.7 μm, 2.1 × 50 mm) maintained at 40°C. The flow rate was 0.5 mL/min using 0.1% formic acid in water and 0.1% formic acid in acetonitrile as mobile phase A (MA) and B (MB), respectively. Initial gradient was set as 93% MA from 0 to 1.0 min; from 1.0 to 13 min, changed from 93% MA to 65% MA; at 13.1 min to 5% MA and maintained at 5% MA until 14 min; then changed to 93% MA at 14.2 min through the end of the run. Data was processed by Masslynx Analyst 4.2 (Waters, Milford, MA). Calibration curve was fitted using peak area ratio of LQG/SIL-LQG with the linear regression and 1/x weight factor. The lowest concentration of C5 reference standard (3.9 μg/mL) was within the dynamic range of the assay and was defined as the assay’s LLOQ.

For analysis of total C5 in mouse serum, standards were prepared in serum from in house-generated C5 knock-out mice, in which the mouse C5 gene was deleted (C5^-/-^). Serum from C5^-/-^ mice was also used as a negative control (blank). The tryptic peptide LQGTLPVEAR derived from C5 was detected and quantified by LC-MRM-MS using a Waters Xevo TQ-S with ACQUITY UPLC system at the flow rate of 0.6 mL/min. The rest of procedure is as described for monkey serum samples.

### PK/PD study in cynomolgus monkey

Male, cynomolgus monkeys (n = 4) were stratified based on initial C5 concentrations. Monkeys received a single IV bolus injection of 15 mg/kg of anti-C5 antibody REGN3918, H4H12161P or H4H12170P on day 0. Blood samples were collected from each animal at 0 (predose), 5 minutes, 4 and 8 hours, 1, 2, 3, 5, 7, 10, 14, 18, 21, 28, 35, 42, and 49 days post dose. All blood was processed into serum. Monkey study was performed at Covance Laboratories, Inc.

### Concentration of total C5 by ELISA for stratification of cynomolgus monkey in PK/PD study

Pre-study concentrations of C5 in cynomolgus monkey serum were measured using a commercial enzyme- linked immunosorbent assay kit (Complement C5 Human ELISA Kit ab125963, Abcam, Cambridge, MA) according to manufacturer’s recommendations.

### Total hIgG concentrations for cynomolgus monkey PK/PD study

Total antibody concentrations of anti-C5 antibodies in monkey serum were measured using a direct enzyme-linked immunosorbent assay (ELISA). Microtiter plates were coated with an in-house generated mouse anti-human IgG1/IgG4 Fc mAb. REGN3918, H4H12161P and H4H12170P were then captured on the plate and detected using an in-house generated biotinylated mouse anti-human IgG4 Fc mAb, followed by NeutrAvidin conjugated with horseradish peroxidase (NeutrAvidin HRP). A luminol based substrate specific for peroxidase was then added to achieve a signal intensity that is proportional to the concentration of total REGN3918, total H4H12161P and total H4H12170P. The lower limit of quantitation (LLOQ) is 0.078 μg/mL in neat monkey serum.

### Hemolysis lysis assays for cynomolgus monkey or C5^hu/hu^ mice PK/PD studies

For hemolysis assays, sheep and rabbit red blood cells were obtained from Colorado serum (cat#31112 and 31081, respectively). The hemolytic activity in serum samples from C5^hu/hu^ mice or cynomolgus monkey following SC injection of REGN3918, in-house eculizumab or IgG4^P^ control antibody was evaluated *ex vivo* using a hemolysis assay. Hemolysin-sensitized sheep red blood cells (SRBCs) were used for the classical complement pathway initiated hemolysis assay. Briefly, SRBCs were washed and resuspended at 1x10^9^ cells/mL in gelatin veronal buffer with CaCl_2_ and MgCl_2_ (GVB^++^). To sensitize the SRBCs, cells were incubated with 0.75 mg/mL hemolysin at 37°C for 20 minutes. Sensitized SRBCs were further diluted in GVB^++^ to 2x10^8^ cells/mL. Sera from C5^hu/hu^ mice were used at a 1:10 final dilution and were *ex vivo* supplemented with hC3 at final concentrations of 90 μg/mL. Monkey sera were diluted at 1:20 final dilution. A total of 1x10^8^ sensitized SRBCs in a volume of 100 μL were plated into round bottom 96-well plates followed by addition of 100 μL of diluted test serum. Sensitized SRBCs without addition of test serum (GVB^++^ only) were included to determine background hemolysis, or were lysed by addition of distilled water to determine maximal hemolysis signal. Cells were gently mixed and incubated at 37°C for 10 or 60 minutes. Cells were pelleted by centrifugation at 1,250xg at 4°C for 7 minutes. A total of 100 μL of the supernatant was transferred to a fresh 96-well flat bottom plate and the OD412 was read on a Spectramax microplate reader. The hemolytic activity was calculated as follows:

$$\%\;hemolysis=\frac{test\;sample\;hemolysis\;(OD412)-background\;hemolysis\;(OD412)}{maximal\;hemolysis\;(OD412)-background\;hemolysis\;(OD412)}x\;100$$

Rabbit RBCs (RbRBC) were used for the alternative pathway initiated hemolysis assay. In brief, RbRBCs were washed and resuspended at 2x10^8^ cells/mL in gelatin veronal buffer with 40mM MgCl_2_ and 40mM EGTA (GVB-Mg^2+^/EGTA). A total of 2x10^7^ RbRBCs in a volume of 100 μL were plated into each well of 96 well round bottom plates followed by addition of 100 μL of test serum (final 10%). RbRBCs without addition of test serum (GVB Mg^2+/^EGTA only) were included to determine background hemolysis. The maximal hemolysis signal was determined by addition of distilled water to lyse the cells. Cells were gently mixed and incubated at 37°C for 60 minutes. Subsequently, hemolysis was quantified as described above.

### Determination of C5a levels by ELISA for in vitro CP hemolysis assay

C5a protein levels were determined in supernatants from the CP hemolysis assay using complement-preserved normal human serum (NHS) pre-incubated with REGN3918 or IgG4^**P**^ control antibody. C5a protein levels were measured using the MicroVue C5a ELISA kit according to the manufacturer’s instructions. Briefly, samples were diluted and incubated on plates pre-coated with capture antibody (mouse anti-C5a specific for a neo-epitope on human C5a). Human C5a protein provided by the manufacturer was used as a standard for calibration. C5a in the supernatants was detected by horseradish peroxidase (HRP)-conjugated detection antibody (mouse monoclonal antibody to the C5a region of C5). The chromogenic HRP-substrate, 3,3',5,5'-tetramethylbenzidine, was added to detect HRP activity. A solution of 1N hydrochloric acid was used to stop the reaction, and the optical density at 450nm (OD_**450**_) was measured on a SpectraMax plate reader. Data were analyzed using nonlinear regression (4-parameter logistics) in GraphPad Prism. C5a concentration was expressed as ng/mL of supernatant.

### Restoring hemolysis in humanized C5 mouse serum by supplementing with human C3

SRBCs were sensitized with hemolysin in GVB^++^, followed by incubation for 60 minutes at 37°C with 2-fold serially diluted test sera (1:10 to 1:640) to initiate hemolysis via the CP. Complement-preserved sera from at least 5 WT or *C5*^*hu/hu*^ mice each were pooled for this analysis. Serum from *C5*^*hu/hu*^ mice was supplemented with human C3 at 40, 80, and 120 μg/mL in the 1:10 dilution followed by 1:2 serial dilution (final 0.625, 1.25, and 1.875 μg/mL in the 1:640 dilution).

### A4F-MALLS analysis

In-house eculizumab was mixed with C5 in 1X DPBS, pH 7.4 and allowed to incubate at room temperature for 1 hour. Then REGN3918 was added to the pre-formed complexes to yield final concentrations of 0.7 μM in-house eculizumab, 0.8 μM C5, and 4.3 μM REGN3918. The final solution was allowed to incubate for an addition 1 hour prior to analysis on an A4F-MALLS system composed of an Eclipse^™^ 3+ A4F Separation System (Wyatt Technology) coupled to an Agilent 1200 Series HPLC system equipped with a ultraviolet (UV) diode array detector, Wyatt Technology Dawn HELEOS® II laser light scattering instrument (LS), and an Optilab^®^ T-rEX differential refractometer (RI) detector.

All the samples were maintained unfiltered at 4°C prior to injection into an Eclipse^™^ short channel fitted with a W350 spacer foil (350 μm spacer thickness, 2.2 cm to 0.2 cm spacer width from the inlet to the outlet, 14.5 cm spacer length) and using a 10 kDa MWCO Nadir regenerated cellulose membrane. The channel was pre-equilibrated with the mobile phase buffer (10 mM sodium phosphate, 500 mM sodium chloride, pH 7.0 ± 0.1), prior to the injection of each sample. The fractionation method consisted of four steps: injection, focusing, elution, and a channel “wash-out” step. Each sample (7 μg of complex sample, and 4 μg of each control sample) was injected at a flow rate of 0.2 mL/min for 1 minute and subsequently focused for 5 minutes with a focus flow rate of 1.0 mL/min. The sample was eluted with a channel flow rate of 1.0 mL/min with the linear gradient cross flow from 2.0 mL/min to 0 mL/min over 45 minutes. Finally, the cross flow was held at 0 mL/min for an additional 5 minutes to wash out the channel. Bovine serum albumin (BSA; 2 mg/mL; 10 μg sample load) was fractionated using the same parameter settings as a system suitability control. Data was collected using ASTRA software (Version 6.1.2 Wyatt Technology) and data analysis was performed using ASTRA software (Version 7.1.0 Wyatt Technology) with an experimentally determined dn/dc value and extinction coefficient at 215 nm (corrected for glycosylation) derived from the protein conjugate analysis provided in the software.

### HDX epitope mapping of REGN3918 binding to C5

HDX-MS was performed on an integrated Waters HDX/MS platform, consisting of a LEAP Tech HDX PAL system (Morrisville, NC), a Waters Acquity M-Class (Milford, MA) and Synapt G2-Si mass spectrometer. For deuterium labeling, 3.8 μL of C5 (EMD Millipore) or C5 premixed with the REGN3918 in 1:1 molar ratio was incubated with 56.2 μL D_2_O labeling solution (10 mM PBS in D_2_O at pD 7.0) for various time-points. The deuteration was quenched by transferring 50 μL sample to 50 μL pre-chilled quench buffer (0.2 M TCEP, 6 M guanidine chloride in 100 mM phosphate buffer, pH 2.5) and then was incubated at 1.0°C for two minutes. The resulting sample was injected into a Waters HDX Manager for online pepsin/protease XIII digestion, trapped onto VanGuard pre-column at 0°C and eluted to ACQUITY BEH C18 1.0 × 50 mm column (130A, 1.7 μm) for a 9-minute gradient separation by 5%-40% MB (mobile phase A: 0.1% formic acid in water; mobile phase B: 0.1% formic acid in acetonitrile). The mass spectrometer was set at cone voltage of 37 V. LC- MS^E^ data were processed and searched against the database including human C5, pepsin, and their randomized sequence via Waters ProteinLynx Global Server (PLGS) software. The identified peptides were imported to DynamX software, which, based on preset criteria, automatically determined deuterium uptake of each peptide across multiple time points based on retention time and mass accuracy (<10ppm).

## Supporting information

S1 Checklist (PDF)

S1 Fig Anti-C5 antibodies block CP and AP complement-mediated hemolysis in normal human and cynomolgus monkey serum *in vitro*. (A-D) Sera from human and monkey were used to examine the efficacy of anti-C5 mAbs to block hemolysis mediated by the classical and alternative complement pathways *in vitro*. The percent block in CP- and AP-mediated hemolysis assays using either human (A, C) and cynomolgus monkey (B, D) serum was determined over a range of concentrations (0.4 to 400nM) for each antibody to generate dose-response curves; REGN3918 is shown in blue. All hemolysis assays are representative experiments that were performed in duplicate at each mAb concentration; each experiment was repeated with multiple sera from different donors (n > 3). Data is plotted as mean ±SD.
(TIFF)

S2 Fig PK/PD properties of anti-C5 antibodies in WT mice, C5hu/hu mice and monkey. (A) Schematic of the strategy used for humanizing the mouse C5 gene (top). A vector containing human genomic C5 sequence, including exons 2–41, partial intron 1, the 3’UTR, and approximately 1.5 kb downstream sequence of the human C5 gene, followed by a neomycin (neo) cassette flanked by loxP sites was used to replace the corresponding region of mouse C5 (middle). The neo cassette was subsequently removed (bottom). (B) Serum C5 levels were determined in *C5*^*hu/hu*^ mice by ELISA, which revealed a difference between male (55.4 ± 1.7 μg/mL, n = 47) and female (24.7 ± 0.6 μg/mL, n = 49) mice. (C) Serum concentrations of antibodies were measured by ELISA at 6 hours, 1, 2, 3, 8 and 14 days post dosing in WT mice administered a single 15 mg/kg subcutaneous dose of anti-C5 mAbs or IgG4^P^ control antibody. REGN3918 is shown in blue. (D) Antibody serum concentrations were measured by ELISA at 2, 4, 7, 10, 14 and 18 days post dosing in *C5*^*hu/hu*^ mice administered a single 15 mg/kg subcutaneous dose of anti-REGN3918 (blue) or H4H12170P (grey). (E-F) RbRBCs in GVB^++^-Mg^2+^/EGTA were incubated for 10 minutes with 5% normal human serum (E) or monkey serum (F), resulting in CP-mediated hemolysis. Pre-incubation of serum with increasing concentrations of REGN3918 or in-house eculizumab blocked hemolysis in human serum a dose-dependent manner. REGN3918 also blocked hemolysis in monkey serum in a dose-dependent manner, but in-house eculizumab failed to block hemolysis in monkey serum. (G) Serum collected from terminally bled *C5*^*hu/hu*^ mice administered with REGN3918 alone (blue), in-house eculizumab alone (red), or in-house eculizumab/REGN3918 switched (green) were supplemented with hC3 and the percent of AP-mediated hemolysis using *ex vivo* assays was assessed. Hemolysis figures are representative experiments that were performed in duplicate at each mAb concentration; each experiment was repeated with multiple sera from different donors (n > 3). Data is plotted as mean ±SD.
(TIFF)

S3 Fig REGN3918 and eculizumab bind to distinct sites on C5 and when all three are present under conditions that are designed to mimic dose switching, form complexes predominantly containing 1 to 2 molecules of C5. (A) The ability of in-house eculizumab to compete with REGN3918 for binding to C5 was examined using real-time, label-free bio-layer interferometry. Binding responses indicating noncompetitive binding of in-house eculizumab and REGN3918 are indicated in bold, whereas competitive binding is highlighted in grey shaded fields. (B) In-house eculizumab:C5 complexes (orange) were analyzed by asymmetric flow field-flow fractionation coupled to multi-angle laser light scattering (A4F-MALLS). Fractograms from individual samples of in-house eculizumab (red), C5 (green), and REGN3918 (blue) are also overlaid. Relative UV absorbance at 215 nm as a function of retention time is shown for each sample and the measured molar masses of resolved peaks are indicated.
(TIFF)

S1 Data (XLSX)
