# Supplementary figures and images for: Inhibition of complement pathway activation with Pozelimab, a fully human antibody to complement component C5
Source: PLoS One. 2020 May 8;15(5):e0231892. doi: 10.1371/journal.pone.0231892 (PMC7209288; doi:10.1371/journal.pone.0231892)

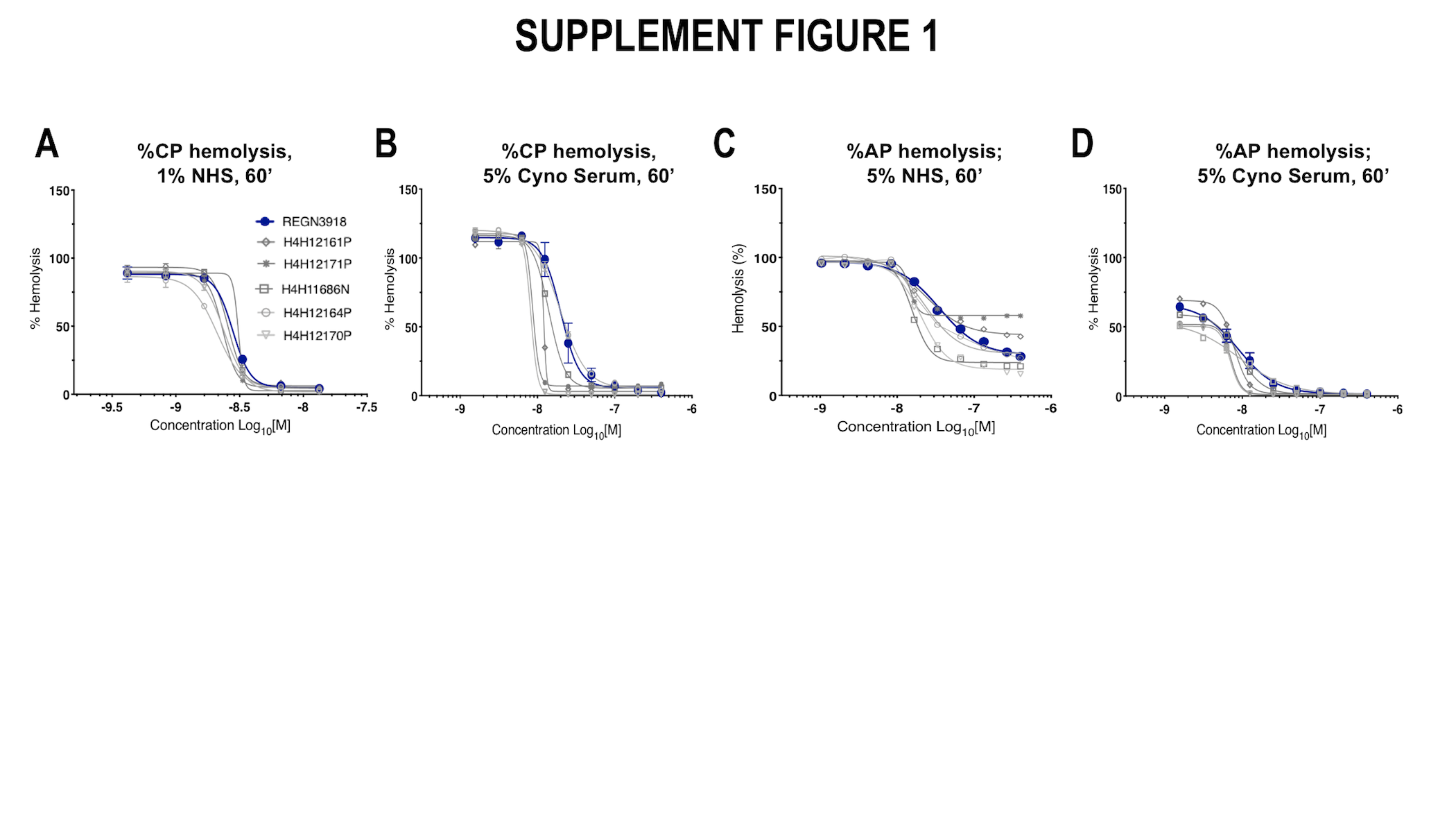

Supplement: S1 Fig — (A-D) Sera from human and monkey were used to examine the efficacy of anti-C5 mAbs to block hemolysis mediated by the classical and alternative complement pathways in vitro. The percent block in CP- and AP-mediated hemolysis assays using either human (A, C) and cynomolgus monkey (B, D) serum was determined over a range of concentrations (0.4 to 400nM) for each antibody to generate dose-response curves; REGN3918 is shown in blue. All hemolysis assays are representative experiments that were performed in duplicate at each mAb concentration; each experiment was repeated with multiple sera from different donors (n > 3). Data is plotted as mean ±SD. (TIFF) [file pone.0231892.s002.tiff]

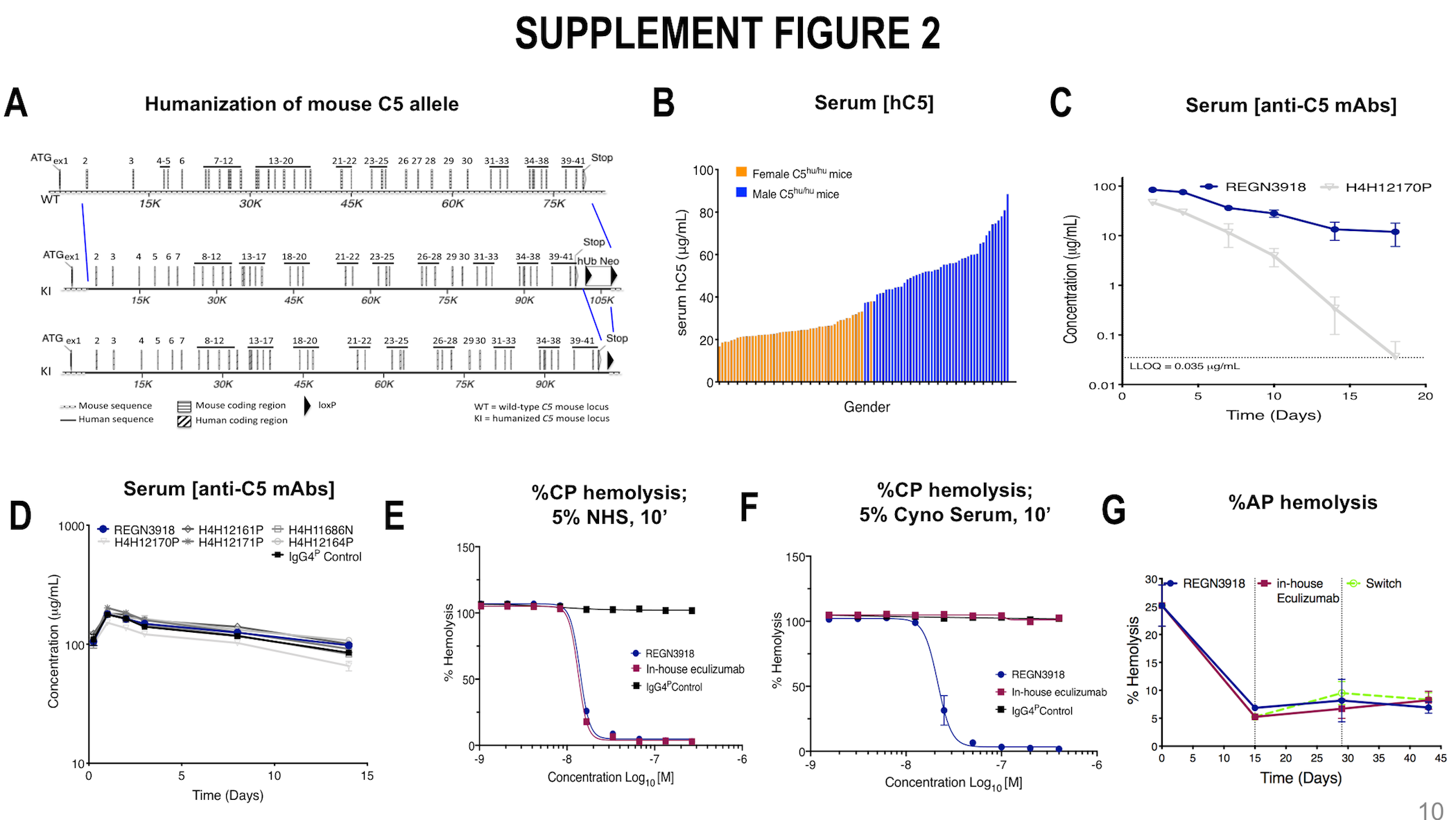

Supplement: S2 Fig — (A) Schematic of the strategy used for humanizing the mouse C5 gene (top). A vector containing human genomic C5 sequence, including exons 2–41, partial intron 1, the 3’UTR, and approximately 1.5 kb downstream sequence of the human C5 gene, followed by a neomycin (neo) cassette flanked by loxP sites was used to replace the corresponding region of mouse C5 (middle). The neo cassette was subsequently removed (bottom). (B) Serum C5 levels were determined in C5hu/hu mice by ELISA, which revealed a difference between male (55.4 ± 1.7 μg/mL, n = 47) and female (24.7 ± 0.6 μg/mL, n = 49) mice. (C) Serum concentrations of antibodies were measured by ELISA at 6 hours, 1, 2, 3, 8 and 14 days post dosing in WT mice administered a single 15 mg/kg subcutaneous dose of anti-C5 mAbs or IgG4P control antibody. REGN3918 is shown in blue. (D) Antibody serum concentrations were measured by ELISA at 2, 4, 7, 10, 14 and 18 days post dosing in C5hu/hu mice administered a single 15 mg/kg subcutaneous dose of anti-REGN3918 (blue) or H4H12170P (grey). (E-F) RbRBCs in GVB++-Mg2+/EGTA were incubated for 10 minutes with 5% normal human serum (E) or monkey serum (F), resulting in CP-mediated hemolysis. Pre-incubation of serum with increasing concentrations of REGN3918 or in-house eculizumab blocked hemolysis in human serum a dose-dependent manner. REGN3918 also blocked hemolysis in monkey serum in a dose-dependent manner, but in-house eculizumab failed to block hemolysis in monkey serum. (G) Serum collected from terminally bled C5hu/hu mice administered with REGN3918 alone (blue), in-house eculizumab alone (red), or in-house eculizumab/REGN3918 switched (green) were supplemented with hC3 and the percent of AP-mediated hemolysis using ex vivo assays was assessed. Hemolysis figures are representative experiments that were performed in duplicate at each mAb concentration; each experiment was repeated with multiple sera from different donors (n > 3). Data is plotted as mean ±SD. (TIFF) [file pone.0231892.s003.tiff]

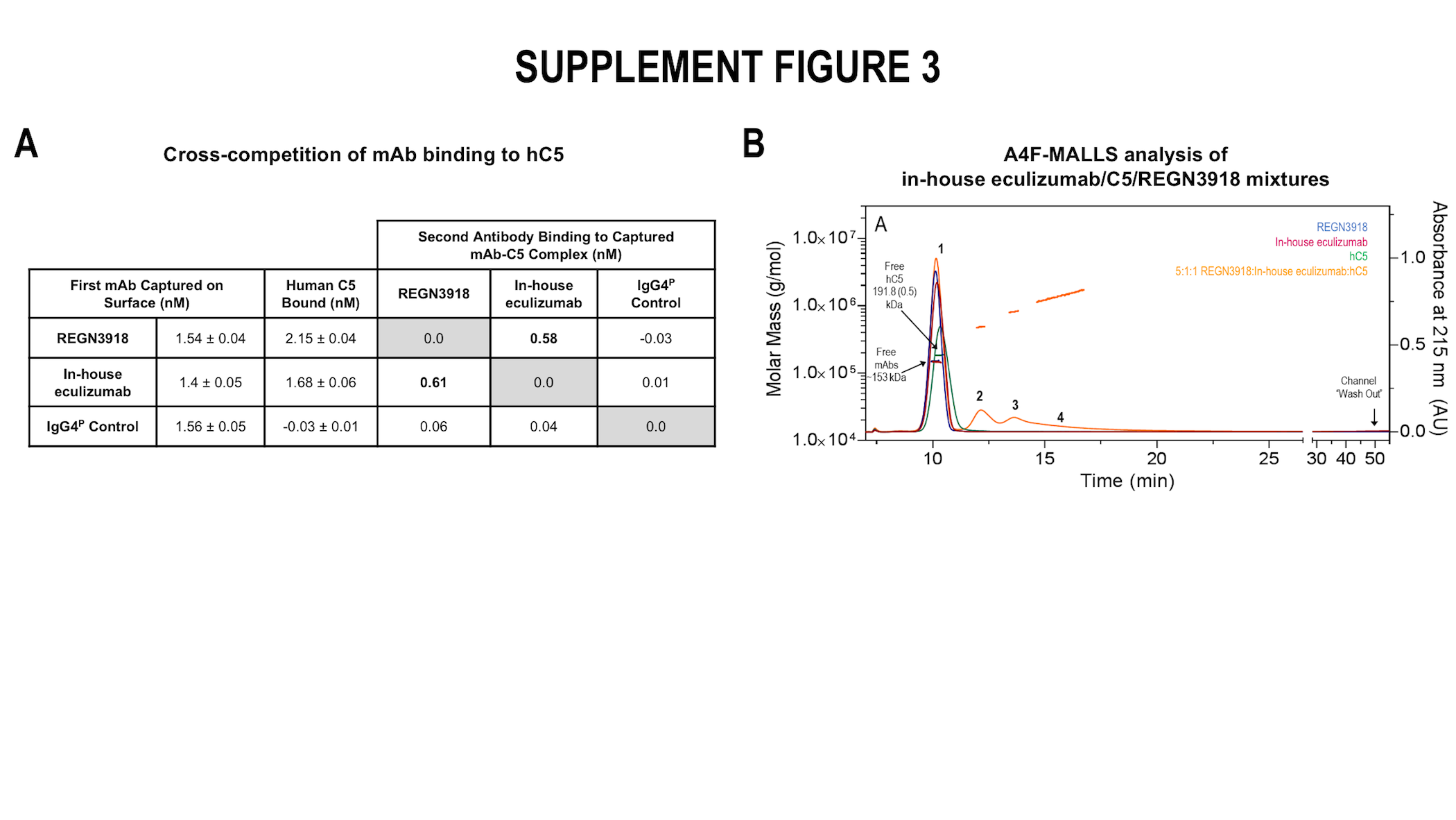

Supplement: S3 Fig — (A) The ability of in-house eculizumab to compete with REGN3918 for binding to C5 was examined using real-time, label-free bio-layer interferometry. Binding responses indicating noncompetitive binding of in-house eculizumab and REGN3918 are indicated in bold, whereas competitive binding is highlighted in grey shaded fields. (B) In-house eculizumab:C5 complexes (orange) were analyzed by asymmetric flow field-flow fractionation coupled to multi-angle laser light scattering (A4F-MALLS). Fractograms from individual samples of in-house eculizumab (red), C5 (green), and REGN3918 (blue) are also overlaid. Relative UV absorbance at 215 nm as a function of retention time is shown for each sample and the measured molar masses of resolved peaks are indicated. (TIFF) [file pone.0231892.s004.tiff]
